# Effect of repeated post-resistance exercise cold or hot water immersion on in-season inflammatory responses in academy rugby players: a randomised controlled cross-over design

**DOI:** 10.1007/s00421-024-05424-3

**Published:** 2024-04-13

**Authors:** Barry G. Horgan, Nicholas P. West, Nicolin Tee, Shona L. Halson, Eric J. Drinkwater, Dale W. Chapman, G. Gregory Haff

**Affiliations:** 1 Australian Institute of Sport (AIS), Australian Sports Commission, Bruce, ACT 2617 Australia; 2 School of Medical and Health Sciences, Edith Cowan University (ECU), Joondalup, WA Australia; 3 Brumbies Rugby, Bruce, ACT Australia; 4 School of Medical Science, Menzies Health Institute QLD, Griffith University, Gold coast, Queensland Australia; 5 Mary MacKillop Institute of Health Research, Australian Catholic University, Melbourne, VIC Australia; 6 School of Behavioural and Health Sciences, Australian Catholic University, Banyo, Queensland Australia; 7 Centre for Sport Research, School of Exercise & Nutrition Sciences, Deakin University, Geelong, VIC Australia; 8 Curtin University, Bentley, WA 6102 Australia; 9 Directorate of Psychology and Sport, University of Salford, Greater Manchester, Salford, UK

**Keywords:** Strength training, Team sports, Hydrotherapy, Recovery, Inflammation

## Abstract

**Purpose:**

Uncertainty exists if post-resistance exercise hydrotherapy attenuates chronic inflammatory and hormone responses. The effects of repeated post-resistance exercise water immersion on inflammatory and hormone responses in athletes were investigated.

**Methods:**

Male, academy Super Rugby players (n = 18, 19.9 ± 1.5 y, 1.85 ± 0.06 m, 98.3 ± 10.7 kg) participated in a 12-week programme divided into 3 $\times$ 4-week blocks of post-resistance exercise water immersion (either, no immersion control [CON]; cold [CWI]; or hot [HWI] water immersion), utilising a randomised cross-over pre-post design. Fasted, morning blood measures were collected prior to commencement of first intervention block, and every fourth week thereafter. Linear mixed-effects models were used to analyse main (treatment, time) and interaction effects.

**Results:**

Repeated CWI (*p* = 0.025, *g* = 0.05) and HWI (*p* < 0.001, *g* = 0.62) reduced creatine kinase (CK), compared to CON. HWI decreased (*p* = 0.013, *g* = 0.59) interleukin (IL)-1ra, compared to CON. HWI increased (*p* < 0.001–0.026, *g* = 0.06–0.17) growth factors (PDGF-BB, IGF-1), compared to CON and CWI. CWI increased (*p* = 0.004, *g* = 0.46) heat shock protein-72 (HSP-72), compared to HWI.

**Conclusion:**

Post-resistance exercise CWI or HWI resulted in trivial and moderate reductions in CK, respectively, which may be partly due to hydrostatic effects of water immersion. Post-resistance exercise HWI moderately decreased IL-1ra, which may be associated with post-resistance exercise skeletal muscle inflammation influencing chronic resistance exercise adaptive responses. Following post-resistance exercise water immersion, CWI increased HSP-72 suggesting a thermoregulatory response indicating improved adaptive inflammatory responses to temperature changes, while HWI increased growth factors (PDGF-BB, IGF-1) indicating different systematic signalling pathway activation. Our data supports the continued use of post-resistance exercise water immersion recovery strategies of any temperature during in-season competition phases for improved inflammatory adaptive responses in athletes.

## Introduction

Athletes engage in resistance exercise across a season, to maintain and develop muscular hypertrophy, strength and power. Each individual resistance exercise session induces a systemic signalling response, which accrues over time following multiple repeated exposures. Athletes also often complete post-resistance exercise recovery strategies, e.g., water immersion, to promote recovery between training sessions. The hydrostatic pressure and temperature effects of water immersion alter the acute post-exercise physiological responses (Petersen and Fyfe 2021), by reducing acute muscle damage and swelling in athlete (Horgan et al. 2022a, b, c). Over time, these acute recovery responses may accumulate to accrue (promote) or mediate (diminish) the chronic systemic inflammatory signalling responses to resistance exercise, (Cornish et al. 2020).

Cold water immersion (CWI) involves the immersion of the body, i.e., single limb, partial- or whole-body (to neck, excluding head), in water temperatures ranging between 8 and 20 °C for a duration range of 5 to 30 min (Moore et al. 2022). The use of CWI throughout a taper period during competition preparation resulted in unclear (15 °C × 15 min, whole-body) (Halson et al. 2014) or positive (15 °C × 15 min, whole-body; 10 °C × 10 min, partial-body) (Halson et al. 2014; Tavares et al. 2020) performance responses, which may be associated with attenuated increases in post-exercise inflammation. If the practice of applying post-resistance exercise CWI (8 °C × 20 min, single-limb; 10 °C × 10 min, partial-body) occurs repeatedly over time, this may result in the accumulation of multiple acute reductions in myofibrillar protein synthesis (Fuchs et al. 2020a, b) and the down-regulation of satellite cell activity and hypertrophy signalling pathways (Roberts et al. 2015). These findings have contributed to scepticism regarding repeated CWI application following resistance exercise in athletes.

Optimising anabolic hormonal status, i.e., testosterone, has also been shown to be favourable for increases in lean muscle mass and strength in healthy males (Bhasin et al. 2001). A single post-resistance exercise hot water immersion (HWI) session (39 °C × 15 min, whole-body) has been shown to result in small acute increases in testosterone concentration in athletes, as compared to a contrast water immersion strategy (Horgan et al. 2022b(in press)). Acute responses to post-resistance exercise HWI application also resulted in increased perceived sleep quality, while reducing next day perceptions of fatigue in athletes (Horgan et al. 2022b(in press)). As such, HWI may offer practitioners an alternative post-resistance exercise strategy to promote positive adaptive responses in athletes. Physiologically, HWI (~ 44 °C × 15–20 min, partial-body) increases peripheral muscular blood flow, as well as skin, muscle and core temperature (Bonde-Petersen et al. 1992). As highly trained athletes are reported to immerse themselves in hot water temperatures of ~ 38–42 °C (Rodrigues et al. 2020; Méline et al. 2021), it remains unclear if this strategy will stimulate positive adaptation (Fuchs et al. 2020a, b; Stevens et al. 2020) when applied repeatedly over time. Hence, the cumulative effects of applying post-resistance exercise CWI versus HWI across multiple sessions on the medium- to long-term hormone responses in athletes warrants further investigation.

Compared to recreationally active subjects, highly trained athletes demonstrate different inflammatory responses to exercise (Damas et al. 2018). Increased training age and status is generally associated with lower exercise-induced muscle damage, e.g., creatine kinase (CK), myoglobin (Damas et al. 2018); adapted increases, e.g., interleukin (IL)-4, IL-6, IL-7, IL-8, IL-15, insulin-like growth factor (IGF)-1, heat shock protein (HSP)-72; or decreases, e.g., IL-1ra, IL-1b,(Dennis et al. 2004) tumour necrosis factor (TNF)-α in inflammatory cytokines and myokines (Cornish et al. 2020), growth factors (Kraemer et al. 2017), and temperature-dependent, muscle regulating HSP-72 (Fyfe et al. 2019). Research investigating repeated post-resistance exercise water immersion in athletes is ambiguous, with few studies highlighting the discrepancies between responses in trained versus untrained subjects (Fyfe et al. 2019). Many studies utilise ‘recreationally active’ subjects, from which results cannot be directly translated to athletes. In studies which utilised highly trained athletes, CWI application occurred following sport-specific training and not following resistance exercise (Ihsan et al. 2021).

To date researchers have not investigated the cumulative effects of repeated post-resistance exercise water immersion on the adaptive inflammatory responses in a highly trained athlete cohort (Roberts et al. 2015). Studies utilising athletes in high performance training settings which investigate the underlying inflammatory adaptive responses to repeated post-resistance exercise water immersion are essential to advance the understanding of the role of post-resistance exercise water immersion, and how it can be periodised across training and competition. This study aimed to determine the cumulative effects following a repeated post-resistance exercise (i.e., 4 weeks @ 2/week) CWI (15 °C × 15 min, whole-body) versus HWI (39 °C × 15 min, whole-body) strategy, compared to CON, on the systemic (blood) inflammatory and hormone responses following a 4-week intervention. We hypothesised that following repeated bouts of post-resistance exercise water immersion that: chronic hormone (testosterone, and testosterone to cortisol ratio) and growth factor (IGF-1, IGFBP-3 and PDGF-BB) responses would increase following all water immersion strategies (i.e., CWI or HWI); post-resistance exercise CWI would attenuate, and HWI would promote increases in blood markers of muscle damage (CK, myoglobin), pro-inflammatory cytokines (IL-1b, IL-6, IL-8, IL-17a and TNF-α) and heat-shock protein (HSP-72); and post-resistance exercise CWI would promote increases in anti-inflammatory cytokines (IL-1ra, IL-4 and IL-10), compared to HWI.

## Methods

### Subjects

Using a repeated measure study design, with conservative inputs of an effect size f = 0.15, alpha = 0.1, 3 groups (CWI, HWI, CON) with an estimated total sample of 24 participants with 3 measures (time) and a correlation among repeated measures of 0.85, the achieved beta (power) is 0.823 (G*Power 3.1.9.2 software). This estimated sample accounts for additional participants to improve the confidence of correlations and to account for the risk of participant drop-out. Thirty-one sub-elite, male, academy Super Rugby players participated. Upon study commencement, subjects were participating in their club adult rugby in-season competition schedule, while also volunteering their participation in this study. In addition to Super Rugby academy and club rugby training, subjects were required to have completed a minimum twice weekly resistance training for > 12 months prior to study commencement. Study approval was obtained from Edith Cowan University and the Australian Institute of Sport (AIS) Human Research Ethics Committees (Approval#:17049). Clinical trial registration was obtained (ANZCTR#:12617000366358), with procedures adhering to the Declaration of Helsinki (2013). Subjects were screened for health and cardiovascular risk using a pre-exercise questionnaire (Adult Pre-exercise Screening System, ESSA, Australia). Subjects were also surveyed regarding any medication or nutritional supplements they were consuming by a qualified sports dietician, prior to confirming suitability to participate in the study. Subjects were informed of study risks and requirements prior to volunteering written informed consent. Prior to study commencement, subjects were familiarised with exercise procedures, post-exercise treatment interventions and measurement test protocols. Subjects were required to complete a minimum of 87.5% (i.e., 7 of 8) of resistance exercise sessions and post-exercise intervention strategies to meet the inclusion criteria for each 4-week block. Subjects were excluded due to injuries sustained during their rugby training or games. Data for eighteen subjects (n = 18, age: 19.9 ± 1.5 y, height: 1.85 ± 0.06 m, weight: 98.3 ± 10.7 kg, body-fat: 18.7 ± 4.1%, body mass index: 28.4 ± 2.5 kg·m^2^, squat jump: 43.1 ± 7.5 cm, counter-movement jump: 45.4 ± 8.4 cm; Yo-Yo Intermittent Recovery Test Level 1: 1560 ± 440 m) were included in the analysis, distributed across CON (n = 17), CWI (n = 17) and HWI (n = 15) interventions.

### Study design

**Fig. 1:**
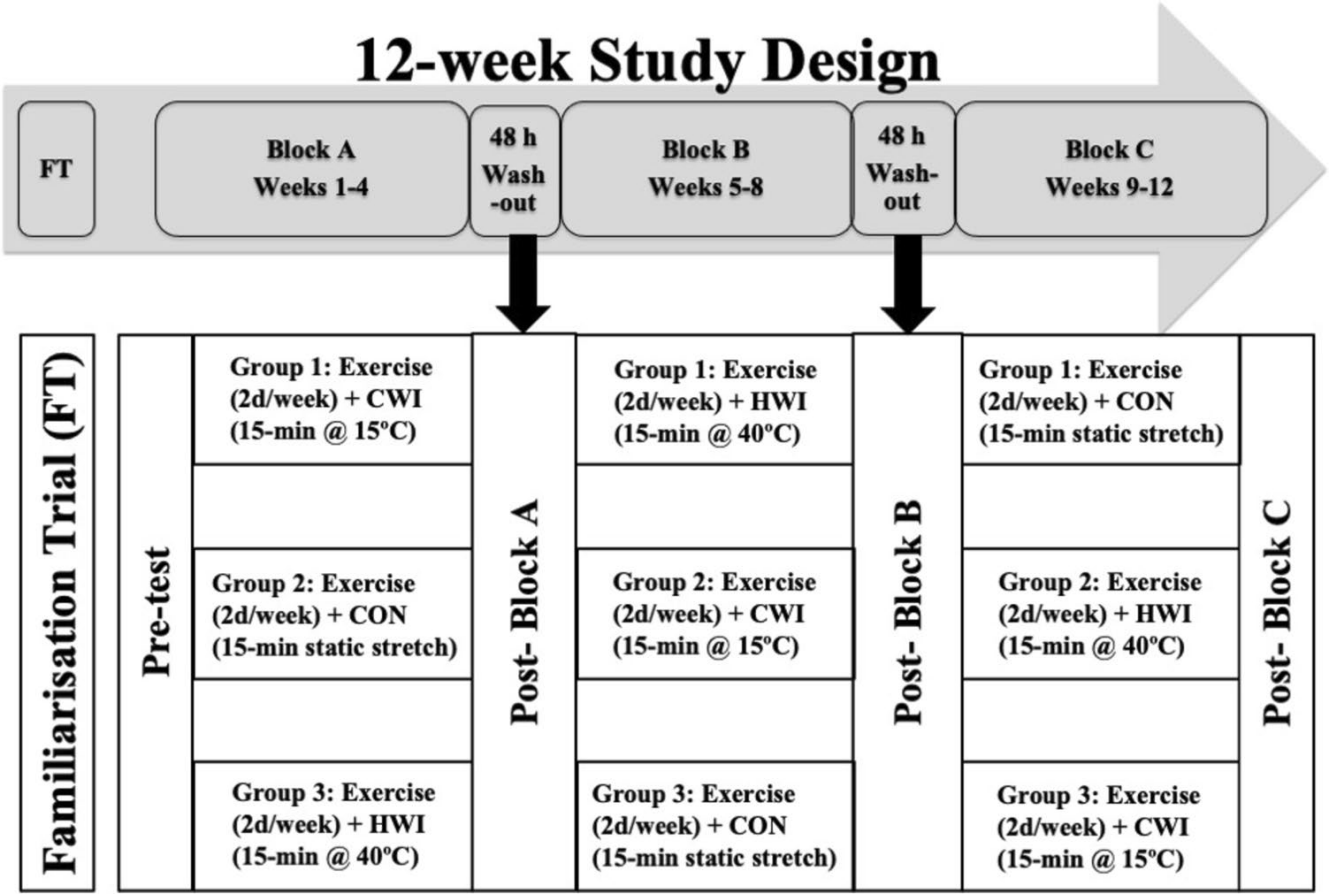
Study design overview

**Fig. 2:**
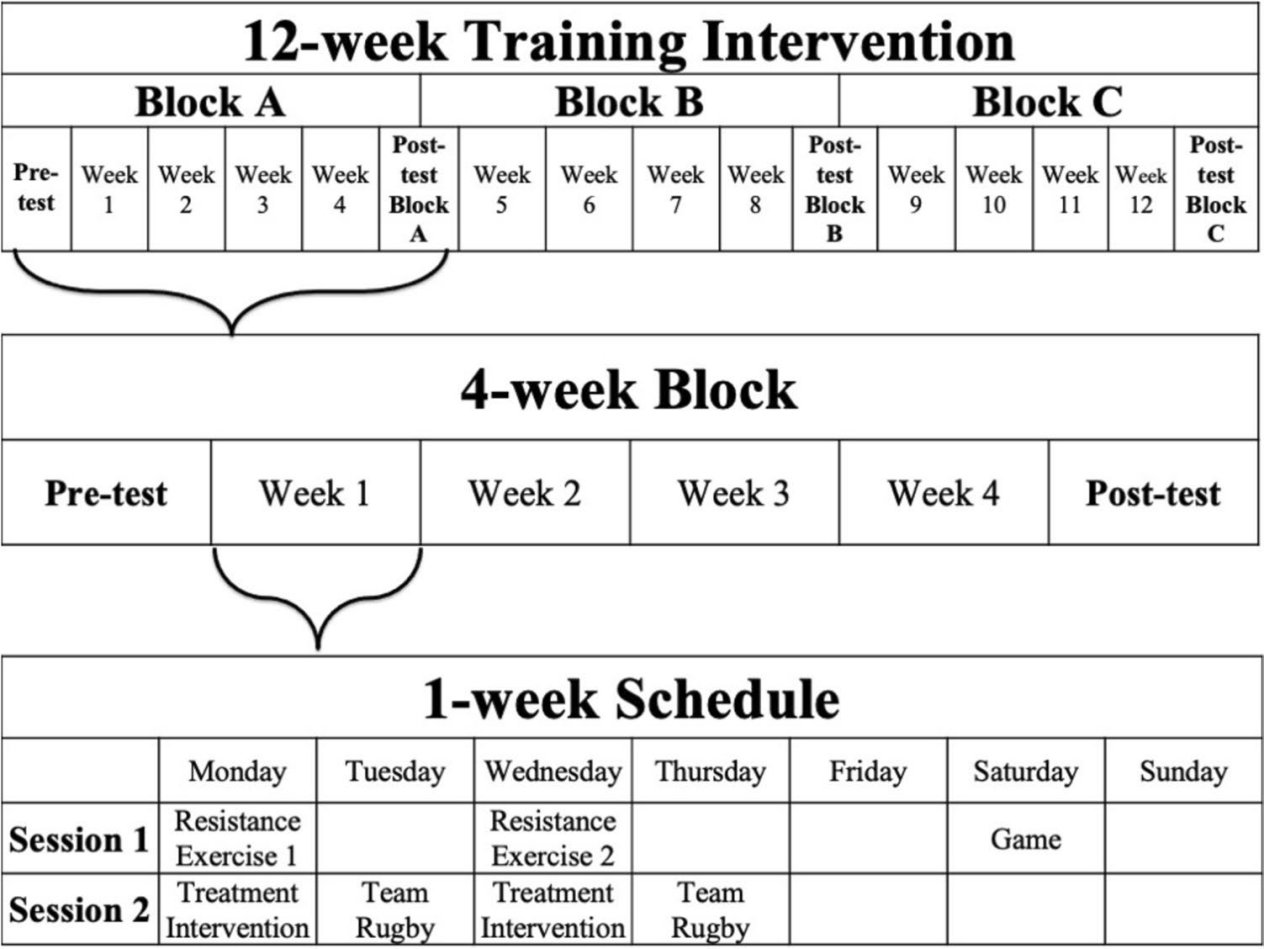
Training intervention schedule overview. Resistance exercise day 1 (Table 1), and day 2 (Table 2), was performed each Monday, and Wednesday respectively, prior to the post-exercise treatment intervention strategy (i.e., CON, CWI or HWI)

A randomised controlled cross-over pre-post design was used, in which subjects undertook a 12-week (4-week $\times$ 3-intervention) programme (refer Figs. 1, 2, (Horgan et al. 2022)). Participants undertook twice weekly resistance exercise sessions, immediately followed by the post-resistance exercise treatment intervention (i.e., CON, CWI, HWI) sessions were performed across each 4-week block (refer Tables 1, 2, (Horgan, et al. 2022a)), for a total of eight resistance exercise and post-resistance exercise treatment intervention sessions each per 4-week block. Resistance exercise was individualised using percentage of body-mass, exercise load-movement velocity, or one repetition maximum. Sessions commenced at the same time of day (i.e., evening), were supervised and performed at room temperature (23–25 °C), with treatment interventions commencing 15-min post-exercise. Training load was monitored using session rating of perceived exertion (sRPE) (McGuigan and Foster 2004). Subjects consumed whey protein isolate (BodyScience, Australia) post-exercise, providing 25 g of protein, 3.2 g of leucine and 5.8 g of branched-chain amino acids. Subjects remained in the same post-resistance exercise intervention strategy, i.e., 15-min CON, CWI (15.1 ± 0.1 °C) or HWI (39.3 ± 0.6 °C) for each 4-week block and crossed-over thereafter. The CON condition performed static stretching at room temperature (23.0 ± 0.6 °C). Subjects in the water immersion strategies sat in the pool, immersed to the neck. Pool water was circulated, with temperatures monitored using a thermometer (Testo AG, Germany). Subjects were advised to avoid showering or bathing for > 2-h after all treatment conditions. Measurement testing occurred following a rest day, immediately prior to commencing the intervention (pre-test) and at the end of each treatment block (i.e., at the end of week 4, 8 and 12), in the morning prior to the subjects’ weekly competitive game. At each testing time-point, fasting, rested morning blood samples were collected, which occurred 36-h following the completion of the last exercise session.

**Table 1:** Description of resistance exercise session 1 completed prior to each post-exercise intervention strategy

Resistance exercise day 1		Week 1	Week 2	Week 3	Week 4	Rest
A. Warm up. LB: SL Squat, OH Lateral leg raise, SL Hip extension; UB: YTW’s, External shoulder raises, SA Push up	Reps	2 × 10	2 × 10	2 × 10	2 × 10	n/a
	% 1RM	n/a	n/a	n/a	n/a	
B1. BB Back squat	Reps	4 × 8	8, 8, 6, 5	6, 6, 5, 3	5, 5, 3, 2	On 4 min turnaround
	% 1RM	66	66, 66, 72, 77	72, 72, 77, 82	77, 77, 82, 87	
B2. Roman chair prone SL Isometric hold	Reps	3 × 30 s	3 × 30 s	3 × 30 s	3 × 30 s	
	% 1RM	n/a	n/a	n/a	n/a	
C1. BB rack pull	Reps	3 × 6	3 × 6	3 × 5	3 × 4	On 4 min turnaround
	% 1RM	65	70	75	80	
C2. Nordic curl	Reps	3 × 4	3 × 4	3 × 4	3 × 4	
	% 1RM	n/a	n/a	n/a	n/a	
D1. BB Bench press	Reps	4 × 8	8, 8, 6, 5	6, 6, 5, 3	5, 5, 3, 2	On 4 min turnaround
	% 1RM	66	66, 66, 72, 77	72, 72, 77, 82	77, 77, 82, 87	
D2. Pull up (prone grip)	Reps	4 × 8	8, 8, 6, 5	6, 6, 5, 3	5, 5, 3, 2	
	% 1RM	66	66, 66, 72, 77	72, 72, 77, 82	77, 77, 82, 87	
E1. DB SA Shoulder press	Reps	3 × 10	3 × 8	3 × 6	3 × 5	On 4 min turnaround
	% 1RM	65	70	75	80	
E2. SA DB Row	Reps	3 × 10	3 × 8	3 × 6	3 × 5	
	% 1RM	65	70	75	80	
F. Core*. *60 s Front plank, 30 s es Side bridge, 60 s Prone bridge	Reps	3 sets each	3 sets each	3 sets each	3 sets each	15 s between sets
	% 1RM	n/a	n/a	n/a	n/a	

*LB* Lower-body, *UB* Upper-body, *OH* Overhead, *SA* Single-arm, *SL* Single-leg, *Reps* Repetitions, *%1RM* Percent of 1 repetition maximum, *BB* Barbell, *DB* Dumbbell, *s* Seconds, *es* Each side, min Minute, *n/a* Non-applicable, 2 × 10: Indicates 2 sets × 10 repetitions were completed; 8,8,6,5: Indicates that a set of 8, 8, 6 and 5 repetitions were performed i.e. a total of 4 sets

**Table 2:** Description of resistance exercise session 2 completed prior to each post-exercise intervention strategy

Resistance exercise day 2		Week 1	Week 2	Week 3	Week 4	Rest
A. Warm up LB: SL Squat, OH Lateral leg raise, SL Hip extension; UB: YTW’s, External shoulder raise and prone press	Reps	2 × 10	2 × 10	2 × 10	2 × 10	n/a
	% 1RM	n/a	n/a	n/a	n/a	
B. 15-min run drilling and accelerations (15-min). 60 s Skipping, A-march, A-skip, A-drill, Accelerations	Reps	3 × 20 m	3 × 20 m	3 × 20 m	3 × 20 m	On 4 min turnaround
	% 1RM	n/a	n/a	n/a	n/a	
C1. Jump monitoring (SJ, and CMJ)	Reps	2 × 5	2 × 5	2 × 5	2 × 5	
	% 1RM	n/a	n/a	n/a	n/a	
C2. BB Jump Squat	Reps	4 × 5	4 × 5	5,5,4,3	4,4,3,2	On 4 min turnaround
	% 1RM	65	70	70, 70, 75, 80	75, 75, 80, 85	
D1. BB Power shrug (floor)	Reps	4 × 5	4 × 5	5,5,4,3	4,4,3,2	
	% 1RM	65	70	70, 70, 75, 80	75, 75, 80, 85	
D2. BB 2-step Step up to Hip-lock	Reps	4 × 4 es	4 × 4 es	4 × 4 es	4 × 4 es	On 4 min turnaround
	% 1RM	30 kg	30 kg	30 kg	30 kg	
E1. 3-way DB Shoulder raise	Reps	3 × 6	3 × 5	3 × 4	3 × 3	
	% 1RM	65	70	75	80	
E2. DB Alternating bench Press	Reps	3 × 10	3 × 8	3 × 6	3 × 5	On 4 min turnaround
	% 1RM	65	70	75	80	
E3. Seated row	Reps	3 × 10	3 × 8	3 × 6	3 × 5	
	% 1RM	65	70	75	80	
F. Core. 12 × Hanging leg raise, 20 × BB Roll-out, 10 es x Lateral pallof press	Reps	3 sets each	3 sets each	3 sets each	3 sets each	15 s between sets
	% 1RM	n/a	n/a	n/a	n/a	

*LB* Lower-body, *UB* Upper-body, *OH* Overhead, *SA* Single-arm, *SL*: Single-leg, *Reps* Repetitions, % 1RM Percent of 1 repetition maximum, *BB* Barbell, *DB*: Dumbbell, *es* Each side, *s* Seconds, min Minute, *N/A* Non-applicable, 2 × 10 Indicates 2 sets × 10 repetitions were completed; 5, 5, 4, 3: Indicates that a set of 5, 5, 4 and 3 repetitions were performed i.e. a total of 4 sets

#### Blood measures

Blood samples were collected via forearm venepuncture to assess systemic blood-based biomarkers (i.e., CK, myoglobin, IL-1ra, IL-1b, IL-4, IL-6, IL-8, IL-10, IL-17a, TNF-α, IGF-1, IGFBP-3, PDGF-BB, HSP-72, testosterone, cortisol) in response to the exercise and intervention. Samples were prepared for cryo-storage and duplicate samples were analysed in the laboratory using methods as previously described (Horgan et al. 2022a, b, c), except for CK. In-season pre-game CK values of 832 ± 812 AU have been reported in high-performance university rugby players and were used as an upper limit (i.e., mean + SD) reference (Mashiko et al. 2004). Inter- and intra-assay CV% were calculated for CK (1.1%, N/A), myoglobin (3.4 ± 3.6, 6.6 ± 6.7%), HSP-72 (5.2 ± 5.1, 8.3 ± 7.1%), IL-1ra (4.0 ± 3.8, 9.9 ± 10.5%), IL-4 (5.1 ± 4.4, 9.3 ± 10.1%), IL-10 (4.6 ± 4.2, 8.9 ± 11.3%), IL-1b (5.8 ± 5.1, 8.2 ± 8.4%), IL-6 (4.3 ± 3.6, 8.5 ± 12.2%), IL-8 (3.4 ± 3.6, 6.6 ± 6.7%), IL-17a (6.2 ± 4.4, 8.5 ± 10.7%), TNF-α (6.5, 2.7%), IGF- 1 (5.3%, N/A), IGFBP-3 (4.8 ± 4.0, 11.7 ± 19.6%), PDGF-BB (8.0 ± 4.7, 12.2 ± 12.1%), testosterone (14.0, 12.0%) and cortisol (9.0, 4.0%). The dynamic range for cortisol and testosterone and is 1000–3,600,000 pg/mL and 0.7–875 nM, respectively.

#### Load monitoring

**Fig. 3:**
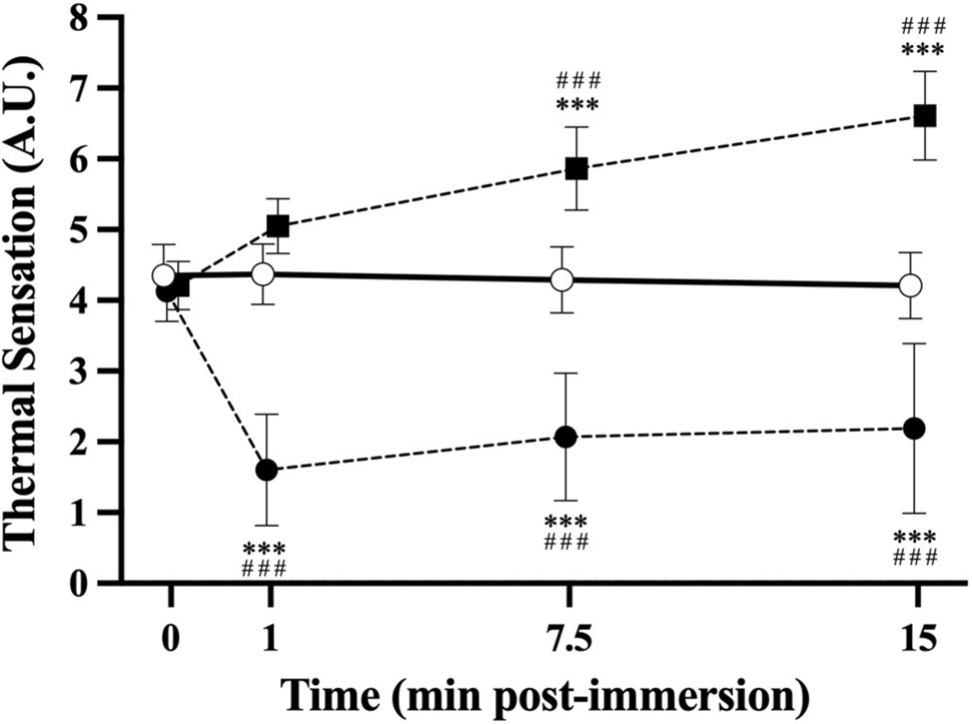
Mean ± SD thermal sensation response over time (min post-immersion) pre- (0 min) and per-immersion (1-, 7.5- and 15-min post-immersion) intervention strategy following whole body resistance exercise with either CON (*line with unfilled* circle n = 17), CWI (*hyphen with filled square* n = 17), or HWI (*hyphen with filled circle* n = 15) intervention. Significant main effects were observed for treatment (*p* < 0.001), time (*p* < 0.001), and treatment × time interaction effect (*p* < 0.001) with Tukey-adjusted *p*-values reporting differences for individual treatment × time pair-wise comparisons at 1-, 7.5- and 15-min following CWI, and HWI, compared to CON condition; ***: *p* < 0.001 versus CON at pre-immersion baseline; ###:*p* < 0.001 versus CON within time-point

sRPE was surveyed for all team training, competition, and resistance exercise sessions (refer Table 3, (Horgan et al. 2022)). Five-day food diaries were collected. Due to challenges with the accuracy of reporting food diary data (Burke 2015), dietician and statistician advice received advised against analysing the diaries due to missing data-points and risk of bias associated with this data collection method. Thermal sensation (TS) (refer Fig. 3, (Horgan et al. 2022)) was assessed for each post-exercise intervention strategy using a 0–8 Likert scale (0 = “unbearably cold”; 8 = “unbearably hot”) immediately prior (0-min), at one minute (1-min), mid-way (7.5-min) and on completion (15-min) of each strategy.

**Table 3:** Descriptive statistics for measures describing the resistance exercise sessions, total weekly training load and post-exercise intervention strategies

Training load variable	CON (N = 17)	CWI (N = 17)	HWI (N = 15)
Resistance exercise session 1 sRPE	316.1 ± 79.5	321.5 ± 101.2	345.5 ± 95.9
Resistance exercise session 2 sRPE	327.5 ± 86.5	324.8 ± 107.6	314.9 ± 82.4
Total weekly sRPE	1574.4 ± 523.7	1591.5 ± 607.9	1789.8 ± 528.9

*sRPE* Session rating of perceived exertion

## Statistical analyses

Descriptive statistics were calculated prior to analysis using software (Stata/IC 15.1, StataCorp LP, USA). Missing data did not show any obvious pattern and were deemed to be at random and no imputation was made. Data were assessed visually via Q-Q plots, as well as using Shapiro–Wilk’s normality test. Outliers were assessed and identified as ‘severe’ if they were ± 3 SD below or above the 25th or 75th quartiles and the authors searched for any obvious reason to exclude these data. Data were analysed using raw values. Linear mixed-effects models (LMM) were conducted to analyse main (treatment, time) and interaction (treatment × time) effects. Subject were set as a random intercept and the model was fitted via maximum likelihood estimation using a 1 × 1 covariance matrix structure. The model analysed treatment intervention (i.e., CON, CWI, HWI) and time (group order, i.e., block A, B, C) as fixed effects on the dependent variable, resulting in nine combinations of treatment and time, with two subjects in each such combination. Significance was set at p ≤ 0.05. Following LMM, main and interaction effects were evaluated using paired samples t-test and corrected using Scheffé’s method of multiple comparison to control for Type 1 error. Intra-class correlations were computed following LMM. Model fit assumptions were assessed via the calculation of residuals. As body composition influences the magnitude of muscle temperature change (Stephens et al. 2017), subject percent of body-fat (BF%) was used as a fixed effect covariate for response variables. Where significant differences were observed between interventions, practical differences in means were interpreted using Hedges g (1981) effect size (ES) statistic, where g $<$ 0.2 = “trivial”, 0.2 $\le$ g $<$ 0.5 = “small”, 0.50 $\le$ g $<$ 0.8 = “medium” and g $\ge$ 0.8 = “large”.

## Results

The treatment groups were balanced for total weekly in-season training load across all rugby and resistance exercise sessions (refer Table 3, (Horgan et al. 2022)). The post-exercise water immersion resulted in significant (time, treatment, and time × treatment interaction effects) changes in TS (refer Fig. 3, (Horgan et al. 2022)), compared to CON.

### Blood measures

#### Muscle damage

**Fig. 4:**
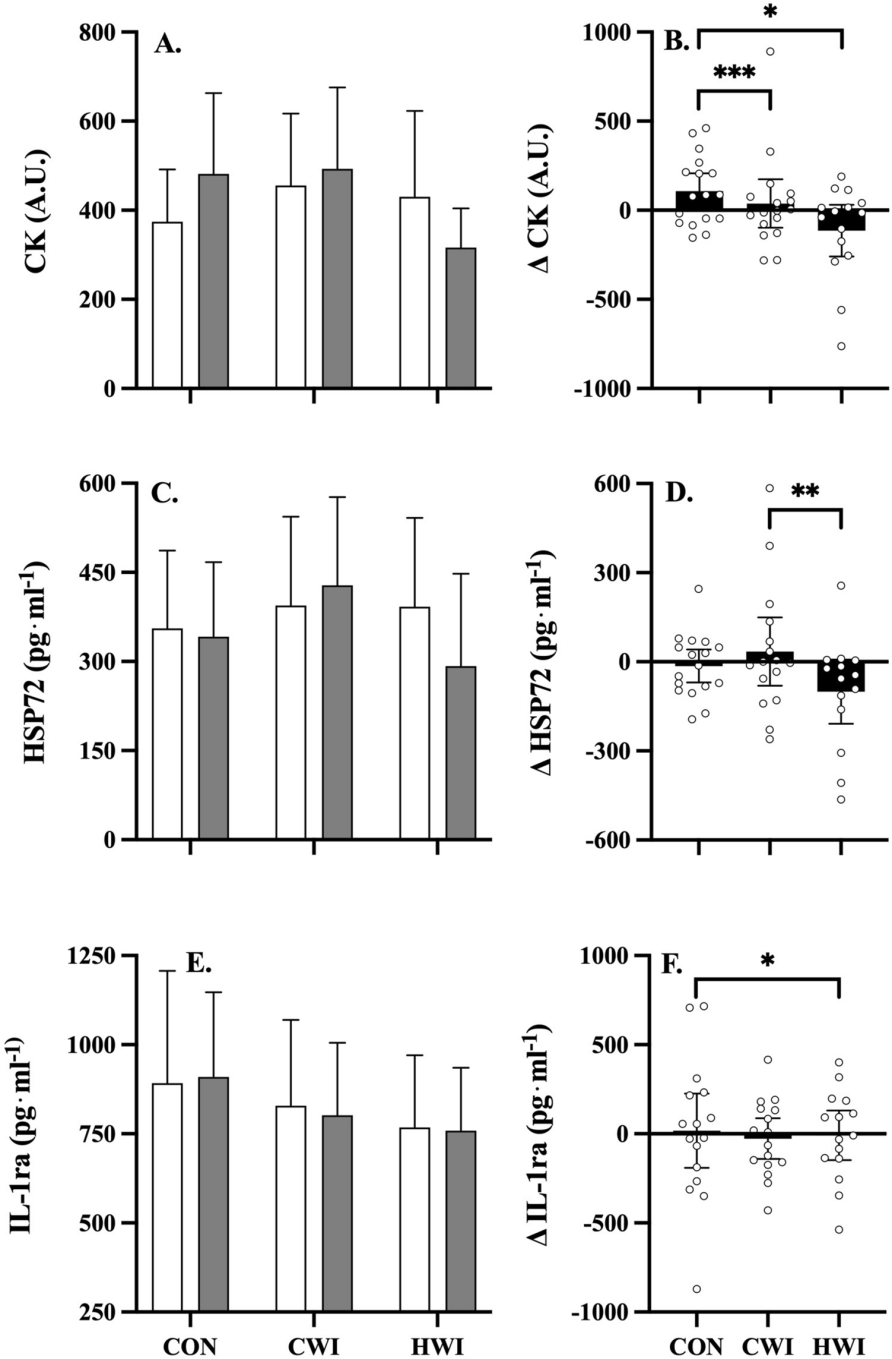
Mean (± 95% C.I.) muscle damage, heat shock protein and anti-inflammatory response pre- (white bar) versus post-intervention (grey bar), and mean (± 95% C.I.) pre- minus post-intervention delta ($\Delta$) (black bar, and individual data points) for CK (panels **A**, **B**), HSP-72 (panels **C**, **D**) and IL-1ra (panels **E**, **F**) following whole body resistance exercise with either control (CON, n = 17), cold (CWI, n = 17) or hot (HWI, n = 15) water immersion as treatment. Significant main effects for treatment were observed in CK (where CON > CWI, *p* = 0.025, *g* = 0.05; and CON > HWI, *p* < 0.001, *g* = 0.62) and HSP-72 (where CWI > HWI, *p* = 0.004, *g* = 0.46); Significant main effects for treatment were observed in IL-1ra (where CON > HWI, *p* = 0.013, *g* = 0.59); *: *p* ≤ 0.05, **: *p* ≤ 0.01, ***: *p* ≤ 0.001

Changes in serum CK concentration are shown in Fig. 4 (Panels A, B). Significant reductions in **Δ**CK (Fig. 4B) occurred following CWI (*p* = 0.025, *g* = 0.05) and HWI (*p* < 0.001, *g* = 0.62), compared to CON. Myoglobin concentration did not differ significantly between interventions (data not shown).

#### Heat shock protein

The effect of post-exercise intervention strategies on serum HSP-72 concentration is shown in Fig. 4 (panels C, D). CWI resulted in significant (*p* = 0.004, *g* = 0.46) increases in HSP-72, compared to HWI.

#### Anti-inflammatory cytokines

Figure 4 (panels E, F) shows the effect of post-exercise intervention strategies on the concentration of serum IL-1ra. CON was associated with significant (*p* = 0.013, *g* = 0.59) increases in IL-1ra, compared to HWI. IL-4 and IL-10 concentrations did not differ significantly between interventions (data not shown).

#### Pro-inflammatory cytokines

Following resistance exercise, the post-exercise intervention strategies did not influence (*p* > 0.05) the concentration of serum pro-inflammatory cytokine concentrations (i.e., IL-1b, IL-6, IL-8, IL-17a, TNF-α; data not shown). Significant positive (IL-8: *p* = 0.001, *r* = 0.22; IL-17a: *p* = 0.029, *r* = 0.26) and negative (TNF-α: *p* < 0.001, *r* = 0.37) associations were observed with the %BF covariate.

#### Growth factors

**Fig. 5:**
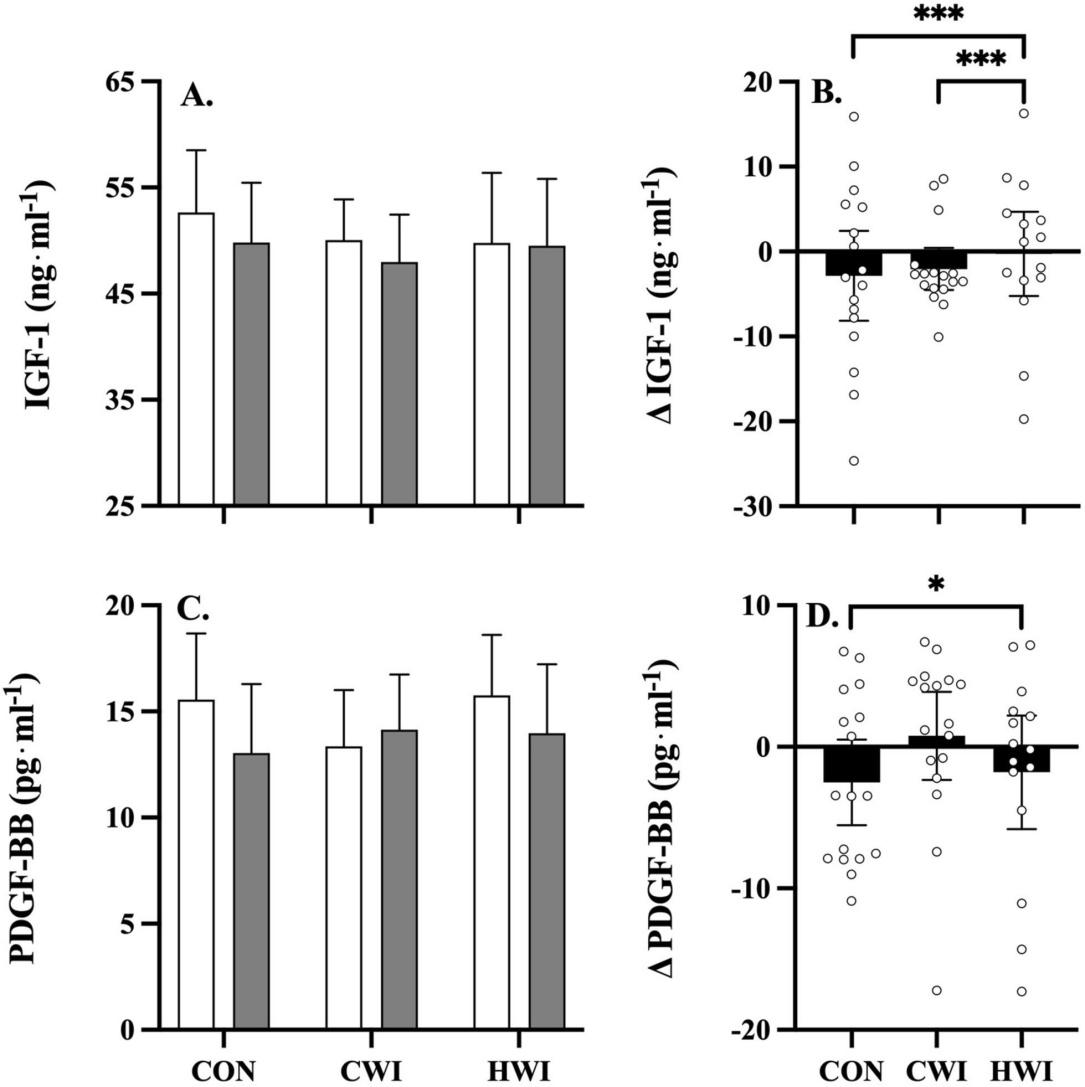
Mean (± 95% C.I.) growth factor responses pre- (white bar) versus post-intervention (grey bar), and mean (± 95% C.I.) pre- minus post-intervention delta ($\Delta$) (black bar, and individual data points) for IGF-1 (panels **A**, **B**) and PDGF-BB (panels **C**, **D**) following whole body resistance exercise with either control (CON, n = 17), cold (CWI, n = 17) or hot (HWI, n = 15) water immersion as treatment. Significant main effects for treatment were observed in IGF-1 (where HWI > CON, *p* = 0.001, *g* = 0.06; and HWI > CWI, *p* < 0.001, *g* = 0.11) and PDGF-BB (where HWI > CON, *p* = 0.026, *g* = 0.17); *: *p* ≤ 0.05, ***: *p* ≤ 0.001

Figure 5 shows the effect of post-exercise intervention strategies on serum growth factor concentrations. IGF-1 (Fig. 5, panels A, B) was significantly lower following CON (*p* = 0.001, *g* = 0.06) and CWI (*p* < 0.001, *g* = 0.11), compared to HWI. PDGF-BB (Fig. 5, panels C, D) was significantly lower following CON (*p* = 0.026, *g* = 0.17), compared to HWI. IGFBP-3 concentration did not differ significantly between interventions (data not shown).

#### Hormones

**Fig. 6:**
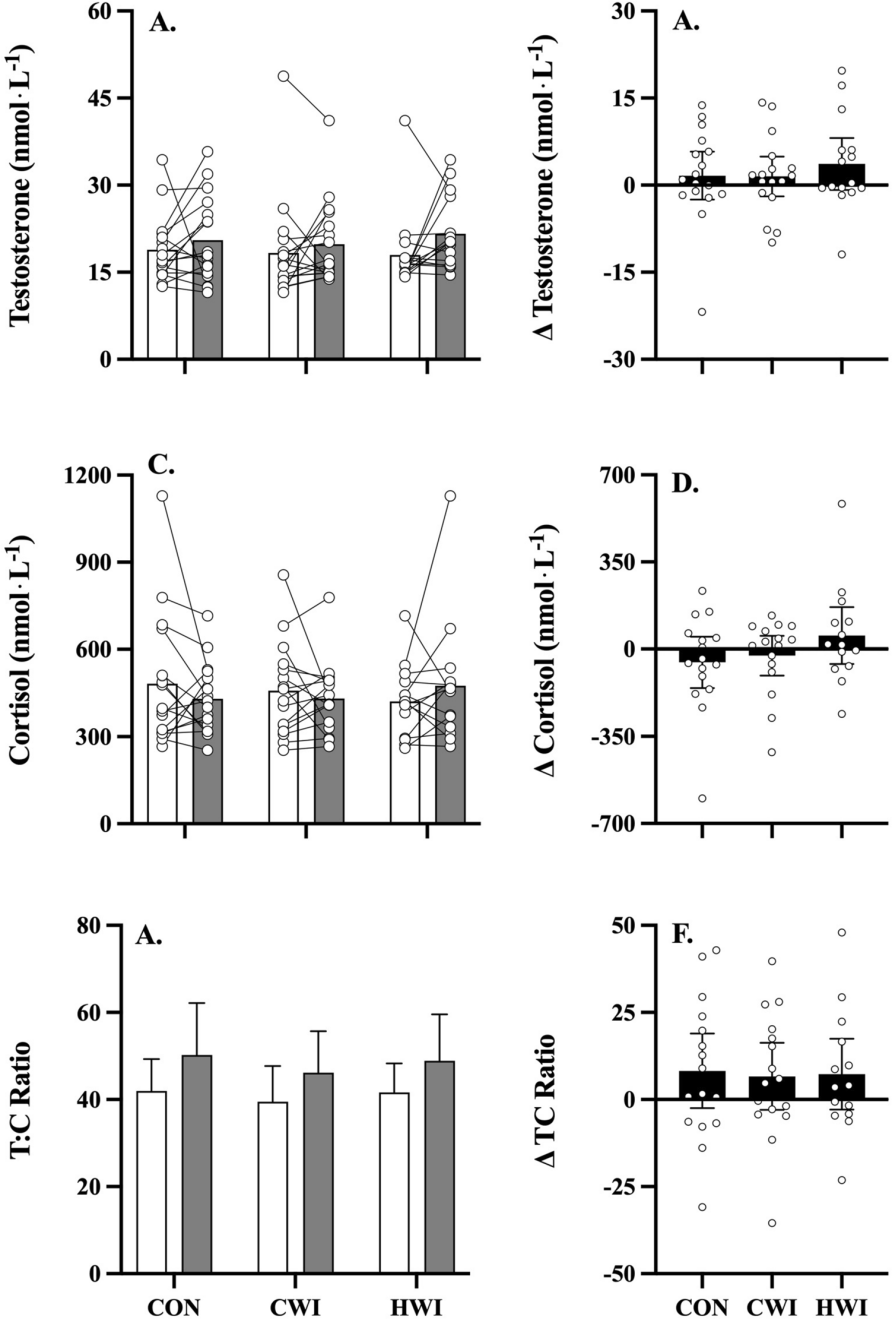
Mean and individual (open circles) hormone responses pre- (white bar) versus post-intervention (grey bar), and mean (± 95% C.I.) pre- minus post-intervention delta (black bar) for testosterone (panel **A**, **B**), cortisol (panel **C**, **D**) and TC ratio (panel **E**, **F**) following whole body resistance exercise with either control (CON, n = 17), cold (CWI, n = 17) or hot (HWI, n = 15) water immersion as treatment. Significant main effects for treatment were observed in testosterone (where HWI > CON, *p* = 0.012); *: *p* ≤ 0.05, **: *p* ≤ 0.01, ***: *p* ≤ 0.001, ****: *p* ≤ 0.0001

Figure 6 shows the effect of post-exercise intervention strategies on serum hormone concentrations.

Testosterone, cortisol, and testosterone to cortisol ratio (TC ratio) concentrations did not differ significantly between interventions (Fig. 6).

## Discussion

During an in-season phase, this study investigated the effects of repeated post-resistance exercise CWI, HWI or CON on inflammatoryresponses in highly trained athletes. We found that repeated post-resistance exercise HWI resulted in moderate reductions in anti-inflammatory cytokine IL-1ra (*p* = 0.013), and muscle damage, i.e., CK (*p* < 0.001); while, HWI also resulted in trivial increases (*p* < 0.001–0.026) in growth factors PDGF-BB and IGF-1, as compared to CON and CWI. Conversely, we found that repeated post-resistance exercise CWI resulted in trivial reductions (*p* = 0.025) in muscle damage, i.e., CK, and small increases (*p* = 0.004) in heat shock protein HSP-72, as compared to HWI. This is one of the first studies to investigate the effects of repeated post-resistance exercise water immersion on an extensive array of inflammatory measures in highly trained athletes.

This study’s findings for muscle damage (i.e., CK) following repeated post-resistance exercise water immersion are consistent with previous research investigating acute responses following a single immersion (Horgan et al. 2022a, b, c), where irrespective of temperature (i.e., cold vs. hot) post-exercise water immersion resulted in trivial-to-moderate reductions in CK, compared to CON. Research to date has predominantly focused on acute effects of post-exercise water immersion. As such, this study is one of the first to investigate the effects of repeated post-resistance exercise water immersion on exercise-induced muscle damage using an all-athlete cohort (Tavares et al. 2018; Méline et al. 2021). The contribution from muscle damage to long-term hypertrophic adaptive responses to resistance exercise is believed to reduce in proportion with increased training status (Damas et al. 2018). Therefore, muscle damage in trained athletes can be considered as non-desirable symptomology associated with resistance exercise. These findings are favourable in terms of the proposed hydrostatic pressure and temperature effects of post-exercise water immersion, which reduces muscle damage biomarkers following resistance exercise. This study observed a large variation in CK, which may be partly explained by other non-modifiable factors, e.g., genetics, ethnicity, gender or age (Scalco et al. 2016). A limitation of the study is the single time-point for blood measures at the end of each 4-week block, given differences in plasma kinetics between biomarkers (Scalco et al. 2016). Future studies may consider examining the longer-term (i.e., > 4-week) pre- versus post-resistance exercise and intervention response of these markers.

Chronic, resting, serum IL-1ra anti-inflammatory cytokine levels were moderately lower (*p* = 0.013) following post-exercise HWI, compared to CON. The chronic effects of resistance exercise on serum cytokines in highly trained athletes remains uncertain. Anti-inflammatory cytokines are thought to play a key role in the inflammatory cascade initiated by damaged muscle from resistance exercise (Methenitis et al. 2021). In our study, the CON group displayed significantly higher muscle damage (i.e., CK), which may be a reason for the elevated IL-1ra when blood testing occurred ~ 36-h after the last session. Similarly, elevated core temperature (~ 39.5 °C) in mice has previously been associated with increased circulating IL-1 levels, providing accelerated recovery and adaptive responses (Dinarello 2012). It should be noted that while there may be some similarities between mice and humans in terms of thermoregulation, there equally are as many challenges in directly comparing both due to the unique thermoregulatory systems of both species (Gordon 2017). For example, humans can take up to 115 min for the rectal temperature to reach 38.8 ± 0.14 °C while sitting passively in HWI at 42 °C (Rodrigues et al. 2020). Future research utilising athletes should investigate the systemic effects that post-exercise water immersion has on acute and chronic responses of genetic and intracellular RNA signalling using closed tube technology, which isolates intracellular RNA, as well as on immune cell subsets using mass cytometry.

Pro-inflammatory cytokines were either positively (i.e., IL-8, IL-17a) or negatively (i.e., TNF-α) associated with percent of body-fat, which was utilised as a covariate in the statistical analysis. This is consistent with findings by Aguiar et al. (2020), where adiposity was positively correlated with pro-inflammatory cytokines in master athletes (Aguiar et al. 2020), with the unexpected exception of TNF-α. A limitation of this study was that we did not provide complete dietary standardisation across the study, with dietary supplementation shown to reduce eccentric muscle damage (Córdova-Martínez et al. 2021) or inflammation markers (e.g., TNF-α) in athletes (Willis et al. 2012). However, our randomised cross-over study design may reduce the effect of this limitation. Another consideration related to nutrition as a covariate for adaptive responses is whether the temperature effects of post-exercise water immersion may influence appetite. Future research should investigate the effects the temperature of post-resistance exercise water immersion may have on markers of appetite regulation.

Post-resistance exercise HWI resulted in trivial increases in IGF-1, compared to CWI or CON strategies, while post-resistance exercise HWI resulted in trivial increases in PDGF-BB, compared to CON. The authors are not aware of any research investigating post-resistance exercise CWI versus HWI in athletes on chronic systemic growth factor responses. Synergistic effects of IGF-1 and PDGF-BB have been reported to provide therapeutic anti-inflammatory osteoarthritic benefits (Montaseri et al. 2011). Collectively, the above results suggest CWI may lower, albeit with trivial effect (*g* = 0.06–0.11), systemic circulating anabolic growth factors, i.e., IGF-1. Our finding is consistent with observations that post-resistance exercise CWI may attenuate the anabolic response in resistance trained males (Earp et al. 2019). In summary, synergistic effects in the systemic circulation of anabolic growth factors (i.e., IGF-1, PDGF-BB) accrue following repeated post-resistance exercise HWI application in athletes, and may contribute towards promoting an anabolic response in athletes, similar to previous studies utilising various forms of heating in combination with exercise stress to enhance adaptation and attenuate atrophy in human and rodent subjects (McGorm et al. 2018; McGorm 2019).

The immersion protocols in the current study reduced (~ 47%) or increased (~ 57%) thermal sensation following CWI or HWI, respectively. Our novel study utilising an all athlete participant cohort is the first to demonstrate small increases occurred in thermo-sensitive serum HSP-72 following repeated CWI, compared to HWI. Our findings are in contrast to evidence demonstrating no change in intramuscular HSP-72 following four weeks of repeated post-endurance exercise CWI (7-week @ 3-day/week, 15 min $\times$ 10 °C) following high-intensity interval cycling exercise in physically active participants (Aguiar et al. 2016), as well as evidence demonstrating that repeated post-resistance exercise CWI (4-week @ 3-day/week, 15 min $\times$ 10 °C) attenuated total intramuscular HSP-72 in recreationally active participants (Fyfe et al. 2019). This unexpected chronic increase in serum HSP-72 following CWI may be explained via a thermal shock-like response due to relative or absolute temperature changes following cooling and upon the homeostatic return to normothermia, i.e., re-warming following a cold shock (Neutelings et al. 2013). Similarly, HSP function and roles differ depending on the tissue of origin (i.e., intracellular versus extracellular), with environmental factors, and exercise mode, duration and intensity all influencing availability (Yamada et al. 2008). It is plausible that our findings are specific to extracellular serum HSP availability following post-resistance exercise CWI in athletes. Given the importance of HSPs in regulating exercise-induced muscular adaptation, this outcome may also partially explain our finding that post-resistance exercise CWI did not attenuate lean muscle mass in athletes. Similarly, reduced resting levels of serum HSP-72 have also been suggested as evidence of an improved inflammatory profile (Hoekstra et al. 2018), as well as evidence of improved heat adaptation via reduced HSP production in response to thermal stressors, e.g., HWI (Gibson et al. 2017).

## Practical applications

Post-resistance exercise water immersion strategies, such as CWI, HWI, etc., may be used to provide trivial-to-moderate reductions in muscle damage (i.e., CK) following a 4-week in-season schedule in highly trained athletes. Water immersion strategies as utilised in this study, may be prescribed for highly trained athletes during an in-season competition phase, posing little risk of attenuating inflammatory adaptative responses. These results support the notion that water immersion may be periodised, i.e., the use of hot increases systemic growth factor circulation (i.e., PDGF-BB, IGF-1), while the use of cold increases thermo-sensitive systemic heat shock protein (i.e., HSP-72) circulation. Practitioners working with athletes should consider individual responses, e.g., as evident by mean ± 95% confidence interval ranges, to post-resistance exercise recovery strategies to identify each athlete’s ‘adaptive sweet spot’. Future study designs should consider the differences in inflammatory responses to exercise and hydrotherapy strategies for trained athletes versus recreationally active, non-athletes.

## Conclusions

This investigation provides insights into systemic inflammatory interactions and responses following post-resistance exercise water immersion. This study provides evidence to inform post-resistance exercise water immersion prescription, i.e., timing and temperature, across a 4-week in-season competition phase for highly trained athletes. Our study, which utilised an athlete cohort, supports the theory of potential differences between athletic and recreationally active participants, which may be partly explained by understanding differing muscle protein synthesis and hypertrophy signalling pathways in trained versus untrained subjects (Damas et al. 2018; Cornish et al. 2020). As athletes become accustomed to resistance exercise, less exercise-induced muscle damage is experienced suggesting that muscle damage is not the primary mechanistic driver for muscular adaptation in athletes (Rossi et al. 2018). It is likely that the synergistic effects of multiple inflammatory responses following resistance exercise trigger both muscle damage and other pro- and anti-inflammatory cytokine, hormone, growth factor, heat shock protein and haematological responses which may cumulatively mediate the downstream muscle protein synthesis and hypertrophy signalling pathways. Future studies should investigate the effect of repeated post-resistance exercise water immersion on other mechanistic adaptive responses in athletes, such as changes in muscle architecture and electromyography.

## Data Availability

The datasets generated and analysed during the current study are not publicly available due to sensitivity associated with high-performance athlete data, however, are available from the corresponding author on reasonable request.

## Abbreviations

CK: Creatine kinase
CON: Control
CWI: Cold water immersion
HSP: Heat shock protein
HWI: Hot water immersion
IGF: Insulin-like growth factor
IL: Interleukin
PDGF: Platelet-derived growth factor
TNF: Tumour necrosis factor
